# Health Literacy Status and Understanding of the Prescription Instructions in Diabetic Patients

**DOI:** 10.1155/2018/4517243

**Published:** 2018-06-11

**Authors:** Shivangini Singh, Sahana Devadasa Acharya, Ashwin Kamath, Sheetal D. Ullal, Rathnakar P. Urval

**Affiliations:** ^1^Department of Pharmacology, Kasturba Medical College, Mangalore, Manipal Academy of Higher Education, Karnataka 575001, India; ^2^Department of Pharmacology, Kanachur Institute of Medical Sciences, Mangalore, Karnataka, India

## Abstract

This study aimed to assess the health literacy (HL) of patients having diabetes mellitus, their understanding of prescription instructions (PI), and the correlation between HL and understanding of PI. A cross-sectional survey was conducted in 263 adult diabetic patients who were assessed for their understanding of route of intake of the prescribed medication(s), frequency of intake, number of medication(s) to be consumed each time, indication for the medication(s), and the relation of drug intake with food. The HL of the patients was assessed by using Rapid Estimate of Adult Literacy in Medicine, a screening test comprising of 66 health-related words. The number of correctly pronounced words was used to assign a grade-equivalent reading level. There was a significant difference in the understanding of PI in patients with low and high HL levels. A significant difference was observed between the mean total score for interpreting PI in patients with 7 or fewer years of education compared with the other groups with a higher educational status (*P* < 0.001). To conclude, diabetic patients with low HL level will have difficulty in understanding PI. Hence, an alternative comprehensive strategy needs to be adopted in clinical practice in these patients to provide them the instructions to take medications properly.

## 1. Introduction

Health literacy (HL) is an individual proficiency that includes the skill to acquire, comprehend, and act on suitable health information [1]. In any health care system, patient involvement plays a key role in effective disease management, especially in cases of lifestyle-related diseases which require extensive ongoing self-care [2]. In diabetics, adequate glycemic control aimed at primary and secondary prevention of diabetic complications is necessary for which the patients need to follow strict dietary restrictions, lifestyle modification, and drug regimen. For adherence to these treatment requirements, patients have to understand the physician's instructions. When compared with patients with adequate HL, those who have limited HL find it harder to comprehend their medical condition and its management [3–6]. Understanding one's medication includes the knowledge of drug indication, required dosage, the frequency of drug intake, and for certain medications, some special instructions [7]. The correlation of HL status with the understanding of prescription instructions (PI) in diabetic patients has not been well studied. Determining the presence of any such potential correlation may help in formulating measures for better disease management. The objective of our study was to assess the HL status of diabetic patients, determine their ability to understand the PI, and study the correlation between the HL status and understanding of PI. We hypothesized that diabetic patients with high HL status would understand the PI better.

## 2. Materials and Methods

A cross-sectional study was conducted in adult patients (>18 years of age) with diabetes mellitus who attended the outpatient department of the tertiary care hospitals affiliated to a medical college in India. The study protocol was approved by the institutional ethics committee. All patients provided a written informed consent prior to participation, and the study was conducted in accordance with the Declaration of Helsinki. Patients with severely impaired vision, hearing problems, and those who had not completed even a single year of schooling were excluded from the study.

After the patients completed their consultation with the physician, their understanding of the PI was assessed. The patients were asked the following questions: what is the route of intake for the prescribed medication(s)? What is the frequency of intake of the medication(s)? What is the number of medication(s) units to be taken each time? Should the medication(s) be taken before, with, or after food? And, what is the indication for the prescribed medication(s)? A correct response received a score of 1, and an incorrect or incomplete response received 0 score.

HL was assessed using Rapid Estimate of Adult Literacy in Medicine (REALM), a screening test comprising of 66 health-related words [8]. It assesses an individual's ability to read health-related words. It helps to estimate the level of HL and, hence, can be used to instruct and educate the patients [9]. The number of correctly pronounced words was used to assign a grade-equivalent reading level. The HL scores were categorized into two groups: 0–60: lower literacy level and 61–66: higher literacy level, assuming that these patients would be capable of reading most of the patient education materials.

Three hundred nineteen diabetic patients were screened, and 263 eligible patients were recruited into the study; the recruitment was completed in 4 months. The sample size of 263 patients was calculated with reference to *P* (proportion of patients correctly inferring one or more label instructions among patients with adequate health literacy) 29% [10], 95% confidence interval (CI), and 80% power. The formula used was *n* = *Z*^2^_*α*_ P (1 − *P*)/E^2^, where *Z_α_* = 1.96 at 95% CI; *P* = 29%; *E* = 0.2 (error % of power).

### 2.1. Statistical Analysis

The analysis of study data was done using Statistical Package for Social Sciences, IBM Corporation, version 16.0. Descriptive statistics like proportion, mean ± standard deviation (SD), or median and interquartile range, wherever suitable, were calculated for the different demographic characteristics. The HL score categories were compared with respect to the various demographic characteristics. One-way ANOVA and independent *t*-test were used to analyze the continuous variables. Chi-square test was used to analyze the categorical variables. A *P* value of less than 0.05 was considered as statistically significant.

## 3. Results

Two hundred sixty-three patients with a prior diagnosis of diabetes mellitus were interviewed for determining their HL status and ability to understand PI. The mean age of the patients was 59.3 ± 11.67 years; 187 (71.1%) patients were males. Based on the REALM scores, patients were grouped into different grade ranges as given in Table 1 [11]. Nine percent of the patients had an HL score in the range of 61–66 (higher literacy level) with a grade equivalent to high school education, 28% had an HL score in the range of 45–60 (marginal literacy level) with a grade equivalent to 7th-8th grade, and 63% had a HL score in the range of 0–44 (low literacy level) with a grade below 6.

No significant difference was found between the patients in the higher and lower HL groups with respect to the demographic features, except for the educational status (*P* < 0.001) (Table 2). Forty-one percent of the patients with lower literacy level had 7 years of schooling or less, and 67% of the patients with higher literacy level were graduates.

**Figure 1:**
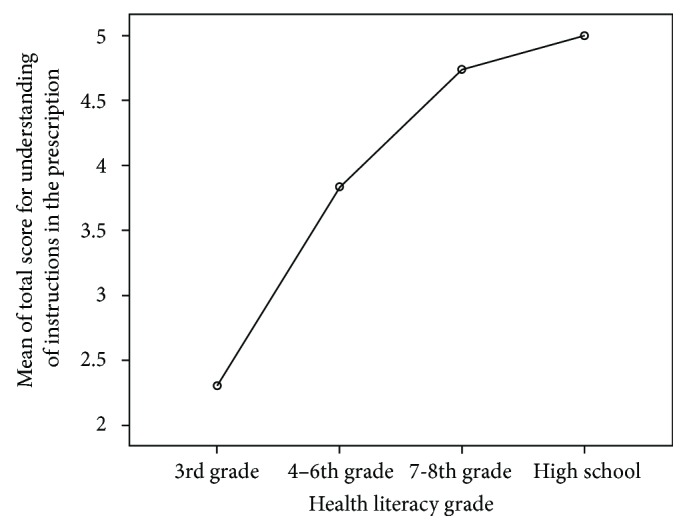
Relationship between understanding of the prescription instructions and health literacy grade among diabetic patients.

**Figure 2:**
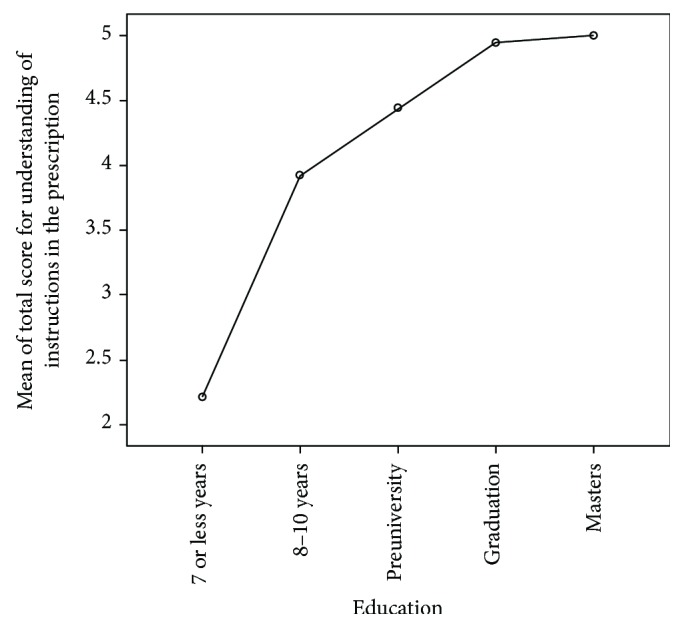
Relationship between understanding of the prescription instructions and education level of diabetic patients.

The educational status and the grade-equivalent HL scores of the study population are shown in Table 3. Out of the 24 patients with HL scores in the range 61–66, 18 were graduates or postgraduates.  Table 4 shows the proportion of patients with lower and higher HL who correctly answered the questions on PI. There was a significant difference (*P* < 0.001) in the understanding of PI between the patients with lower and higher HL levels, with the score for understanding PI increasing with HL level. Among the patients with lower HL, the mean total score for interpreting PI was better in patients with HL grade 7-8 (4.7 ± 0.8) compared with that of patients with HL grade 4–6 (3.83 ± 1.48, *P* < 0.001) and HL grade ≤ 3 (2.31 ± 2.18, *P* < 0.001) (Figure 1). The mean score for understanding PI in patients with 7 or less years of education was (2.21 ± 2.14), which was significantly less compared with the mean score of patients with 8–10 years of education (3.92 ± 1.68, *P* < 0.001), those with preuniversity (4.44 ± 1.26, *P* < 0.001), graduation (4.94 ± 0.23, *P* < 0.001), and master's degree (5 ± 0.00, *P* < 0.046) (Figure 2).

Percentage of patients receiving one medication per prescription was 84.8%, two medications per prescription was 14.4%, and three medications per prescription was 0.76%. The mean total score for interpreting PI was 3.36 ± 2.07, 4.26 ± 1.31, and 5 in patients receiving one, two, and three medications per prescription, respectively. There was a significant difference in mean score for understanding PI between patients receiving one and two medications per prescription (*P* < 0.027). There were only two patients who received three medications per prescription. Patients receiving insulin therapy had a significantly lesser score for interpreting PI when compared with those receiving only oral antidiabetics (3.07 ± 2.16 versus 3.76 ± 1.89; *P* = 0.02). Male patients had a higher score for interpreting PI compared with female patients (3.69 ± 1.87 versus 3.05 ± 2.22; *P* = 0.001). The duration of the disease did not have an effect on the PI interpretation scores.

## 4. Discussion

In this study, we investigated the HL status of diabetic patients and its relationship with the ability to understand PI. Overall, the proportion of patients having low literacy was higher in this study when compared with other studies [7, 9]. Most patients with a higher literacy level were graduates, which suggests that patients with higher educational status have a better HL score than patients with lesser years of schooling as seen in an earlier study [10]. Fifty percent of the graduates also had a lower literacy level, which may be influenced by the medium of education [9]. There was no difference in the literacy levels between males and females in this study. A similar finding was noted in other studies, but it is difficult to draw any conclusion as the proportion of female patients (28.1%) was less in this study [12].

In this study, patients with lower HL had difficulty in understanding all the five questions regarding PI. All the patients in the higher literacy group answered these questions correctly. Seventy-seven percent of the patients in the lower literacy group knew the route of intake of the drug, but the proportion of patients who knew the number of tablets to be taken per dose was the least (62%), which means that the functional HL regarding the prescription medication was poor in diabetic patients who had lower HL scores. These findings might be important in the management of diabetic patients, especially those with lower HL. The findings emphasize the need for adopting specific strategies to improve the understanding of PI. Among the 239 patients with lower literacy level, 117 (49%) answered all the five prescription-related questions correctly which means that HL alone may not influence the understanding of PI. Age and duration of the disease did not influence the understanding of the PI in this study. The lack of age-related difference in the scores for understanding PI could, at least partly, be due to the lesser percentage of patients in the age group of 20–45 years in this study. Unlike in the study by Davis et al., the understanding of PI was better in patients with more number of prescription medications [13].

Our study has limitations. We could not study the impact of HL on the outcome of diabetes treatment because of the cross-sectional study design. The percentage of female patients and those with educational qualification more than preuniversity level was less in this study which could have influenced the findings.

## 5. Conclusion

Diabetic patients with lower HL level will have difficulty in understanding the PI. Hence, an alternative, comprehensive strategy needs to be adopted in clinical practice to provide instructions regarding proper medication use in these patients.

## Figures and Tables

**Table 1:** Scores and grade equivalents for the REALM questionnaire.

Health literacy score	Grade range
0–18	Third grade and below; they will not be able to read most low literacy materials; they will need repeated oral instructions
19–44	Fourth to sixth grade; they will need low literacy materials; they may not be able to read prescription labels
45–60	Seventh to eighth grade; they will struggle with most patient education materials; they will not be offended by low literacy materials
61–66	High school; they will be able to read most patient education materials

**Table 2:** Demographic characteristics of diabetic patients based on their health literacy levels.

Characteristics	Health literacy level		*P* value
	Low (*n* = 239)	High (*n* = 24)	
	Scores 0–60	Scores 61–66	
Age (mean ± SD)	59.04 ± 11.81	61.92 ± 9.93	0.251
Male (%)	170 (71.9)	17 (70.8)	0.976
Female (%)	69 (28.9)	7 (29.2)	
Education (%) 7 years or less	97 (40.6)	1 (4.2)	<0.001^∗^
8–10 years	91 (38.1)	1 (4.2)	
11-12 years	30 (12.6)	4 (16.7)	
Graduation	20 (8.4)	16 (66.7)	
Masters	1 (0.4)	2 (8.3)	
Number of patients on insulin (%)	104 (43.5)	6 (25)	0.08
Number of patients on OHA (%)	147 (61.5)	19 (79.2)	0.087
Number of patients both insulin and OHA (%)	13 (5.4)	1 (4.2)	0.791
Total number of medication per day (mean ± SD)	1.15 ± 0.37	1.21 ± 0.51	0.52
Duration of disease (median (IQR))	8 (4, 13)	10 (5.25, 12)	0.519

SD: standard deviation; IQR: interquartile range; OHA: oral hypoglycemic agents; ^∗^significant association between educational status and literacy level when applied Chi-square test.

**Table 3:** Grade-equivalent health literacy scores and education levels of diabetic patients.

Educational level	HL score (grade equivalent)			
	0–18	19–44	45–60	61–66
	(3rd grade)	(4th–6th grade)	(7th–8th grade)	(High school)
	(%)	(%)	(%)	(%)
Masters	0	0	1 (33.3)	2 (66.7)
Graduation	0	2 (5.6)	18 (50)	16 (44.4)
Preuniversity college	6 (17.6)	5 (14.7)	19 (55.9)	4 (11.8)
8–10 years of schooling	35 (38)	28 (30.4)	28 (30.4)	1 (1.1)
7 or fewer years of schooling	77 (78.6)	13 (13.3)	7 (7.1)	1 (1)

**Table 4:** Correct interpretation of prescription medication instruction by diabetic patients based on their health literacy levels.

Questions	Health literacy level		*P* value
	Low (*n* = 239)	High (*n* = 24)	
	Scores 0–60	Scores 61–66	
For route of administration (%)	183 (76.6)	24 (100)	0.008
Frequency of intake in a day (%)	161 (67.4)	24 (100)	0.001
Number of tablets per dose (%)	148 (61.9)	24 (100)	<0.001
Relation of intake of drug with food (%)	151 (63.2)	24 (100)	<0.001
Drug is for diabetes or other condition (%)	161 (67.4)	24 (100)	0.001

## Data Availability

The data used to support the findings of this study are available from the corresponding author upon request.
